# Bilateral Pneumothorax After Unilateral Subclavian Vein Cannulation for Pacemaker Implantation: A Case Report

**DOI:** 10.7759/cureus.68573

**Published:** 2024-09-03

**Authors:** Takuya Kuninobu, Hirofumi Kawamata, Sakiko Honda, Tatsuya Kawasaki

**Affiliations:** 1 Department of Cardiology, Matsushita Memorial Hospital, Moriguchi, JPN

**Keywords:** pneumothorax, pacemaker, implantation, complication, bilateral

## Abstract

Ipsilateral pneumothorax is a common complication of pacemaker implantation due to transvenous lead placement. We report a case of bilateral pneumothorax after unilateral subclavian vein cannulation for pacemaker implantation. An 85-year-old woman underwent dual-chamber pacemaker implantation for symptomatic atrioventricular block. The transvenous leads were inserted through the left subclavian vein under fluoroscopy and contrast venography, and bilateral pneumothorax was noted the day after implantation, although echocardiography and computed tomography showed no pericardial effusion or lead extrusion. A chest tube was placed in the left chest cavity, which was removed one week later without complications.

## Introduction

Pneumothorax is a well-recognized procedure-related complication of pacemaker implantation with an incidence of 0.9-1.2% because transvenous lead placement requires venous puncture in the prepectoral region [1,2]. We report a case of bilateral pneumothorax after unilateral subclavian vein cannulation for dual-chamber pacemaker implantation.

## Case presentation

An 85-year-old woman presented to the emergency department of our hospital with intermittent dizziness. The patient had been in a normal state of health until approximately one week before presentation, when exertional dyspnea gradually developed. She had hypertension, which was well controlled with amlodipine 2.5 mg daily, olmesartan 20 mg daily, and azelnidipine 16 mg daily. She did not smoke or drink and had no known allergies. There was no family history of cardiovascular disease. On examination, the blood pressure was 177/65 mmHg, the heart rate was 52 beats per minute, the respiratory rate was 16 breaths per minute, and the oxygen saturation was 98% while breathing ambient air. Systolic ejection murmurs were heard, and there was mild edema in the lower legs. The remainder of the examination was normal.

**Figure 1:**
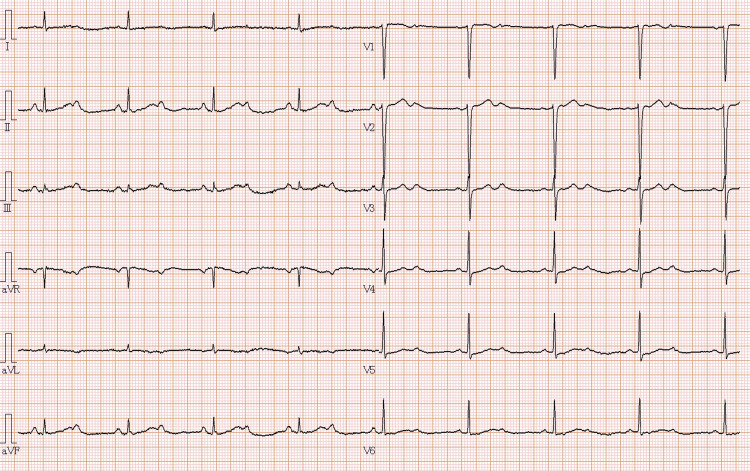
Electrocardiography Electrocardiography on admission shows a second-degree atrioventricular block with 2:1 conduction.

Electrocardiography revealed a second-degree atrioventricular block with 2:1 conduction (Figure 1). A chest radiograph showed no pulmonary congestion, pleural effusion, pneumothorax, or apical bullae. The routine blood tests were normal, as were the thyroid function tests. The brain natriuretic peptide level was 34.7 pg/ml (reference value: ≤18.4). Echocardiography showed a left ventricular ejection fraction of 75% and normal chamber sizes without significant valvular disease. Temporary pacing was deferred given her asymptomatic status at rest. The patient was admitted to the high-care unit for close monitoring.

**Figure 2:**
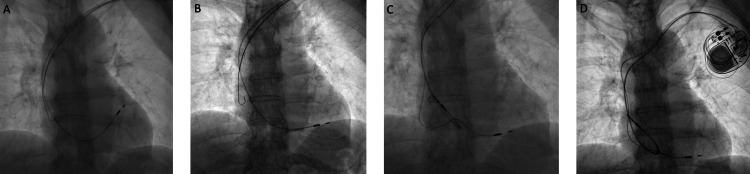
Pacemaker implantation No suitable position for a ventricular lead was found at the mid-to-high intraventricular septum (A). The lead is screwed to the inferior position of the septum (B). The atrial lead is screwed to the right atrial appendage (C), and a generator is connected to the leads (D).

**Figure 3:**
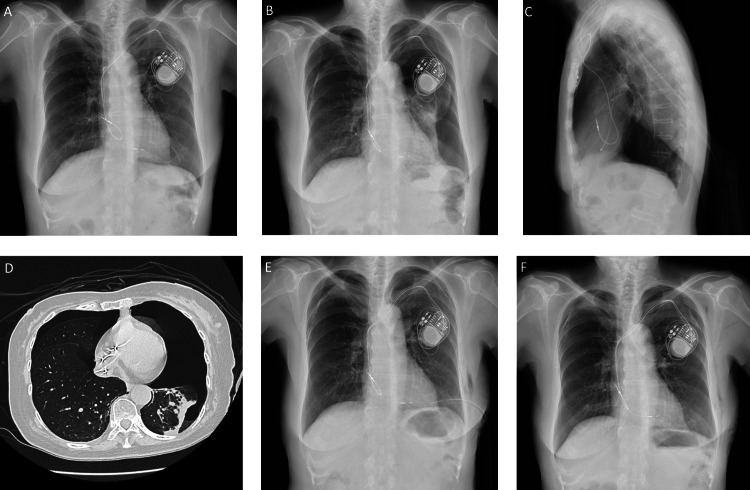
Serial changes in chest imaging Chest radiograph showed no pneumothorax 20 minutes after implantation (A), but a left-sided dominant bilateral pneumothorax is noted (B-D). A follow-up chest radiograph obtained three days after the insertion of the chest tube to the left chest shows improvement of pneumothorax in severity on both sides (E). The next day after the removal of the chest tube, only a mild pneumothorax is shown on the right side (F).

Two days later, she developed a third-degree atrioventricular block and underwent dual-chamber pacemaker implantation via the left subclavian vein under local anesthesia. The left subclavian vein was cannulated using the Seldinger technique under fluoroscopy and contrast venography, and two helical active fixation leads were inserted into the right atrium and ventricle using a 9 Fr peel-away sheath. The introducer needle did not cross the midline of the chest and right subclavian cannulation was not attempted. The ventricular lead was screwed to the inferior intraventricular septum, as a stable position was not found at the mid-to-high level of the intraventricular septum (Figure 2A, 2B). The atrial lead was screwed to the right atrial appendage (Figure 2C). During the procedure, she noted mild chest discomfort, which was treated with intravenous hydroxyzine 50 mg. The vital signs were stable with no chest discomfort at the end of the implantation. No pneumothorax was noted on fluoroscopy (Figure 2D) and chest radiograph 20 minutes after implantation (Figure 3A).

Epigastralgia developed approximately six hours after surgery. Electrocardiography showed normal sinus rhythm without atrioventricular block, right bundle branch block, and no ST-T changes. Acetaminophen 500 mg was started orally and her symptoms resolved. The next morning, chest radiography unexpectedly revealed bilateral pneumothorax (Figure 3B, 3C). Echocardiography and computed tomography of the chest showed no pericardial effusion or extrusion of the leads (Figure 3D). A chest tube was inserted into the left thoracic cavity; the pneumothorax of the right thoracic cavity was considered mild to moderate in severity and tube insertion was waived. The subsequent clinical course was uneventful (Figure 3E), and the chest tube was removed without complication one week later (Figure 3F). The patient was discharged home in stable condition and scheduled for an outpatient pacemaker visit.

## Discussion

The present patient underwent dual-chamber pacemaker implantation for symptomatic atrioventricular block. The transvenous leads were inserted via the left subclavian vein under fluoroscopy and contrast venography, and bilateral pneumothorax was demonstrated the day after implantation. A chest tube was placed in the left thoracic cavity only, as the right thoracic pneumothorax was considered mild to moderate in severity. The subsequent clinical course was uneventful, and the chest tube was removed one week later without complications.

Complications associated with pacemaker implantation can be categorized as immediate, intermediate, and late, with rates ranging from <1% to 6% [1]. Immediate or procedure-related complications include pneumothorax (0.9-1.2% incidence), cardiac perforation (<1%), hemothorax (<1%), pocket hematoma (3.5%), right-sided lead dislodgement (1.8%), venous thrombosis and obstruction (1-3%), device infection (1-1.3%), and mechanical lead complications (<1%) [1]. Post-operative chest radiographs, including lateral or oblique views if necessary, and electrocardiography are routinely performed to detect these complications, neither of which were informative in our patient. Echocardiography and computed tomography of the chest after implantation may also be useful for the early detection of procedure-related complications, although not recommended in all cases.

In the current patient, bilateral pneumothorax was confirmed the day after implantation. It is reasonable to assume that the left pneumothorax was associated with a puncture of the left subclavian vein, as ipsilateral pneumothorax is one of the most common complications during the procedure [1]. To reduce the risk of pneumothorax, vascular access using anatomical landmarks such as venous visualization with contrast venography [3], as in our case, or ultrasound guidance [4] as well as extrathoracic venous puncture may be recommended. Of note, the current patient developed a contralateral pneumothorax despite not attempting a right-sided approach. The exact incidence of bilateral or contralateral pneumothorax remains unclear, although there are anecdotal reports [5-9].

One proposed mechanism for contralateral pneumothorax is lead perforation [5-7], although considered extremely rare (i.e., estimated <0.0001% of all pacemaker implantations) [5]. In the current case, we cannot completely exclude the possibility of electrode perforation of the atrial wall, pericardium, and pleura, although there was no convincing evidence of extrusion of the helix of the screw-in atrial lead. In general, the perforation appears to be small and spontaneously occluded and occurs while searching for a good atrial position [5]. It is reported that in a prospective observational study of 968 consecutive patients undergoing implantation or upgrade, pericardial effusion was common, although rarely requiring therapy [10].

Intrapleural communication, also known as buffalo chest, may be another cause of bilateral pneumothorax; this condition may be congenital or may develop after cardiothoracic and mediastinal surgery or trauma [8,9]. The current patient did not have findings consistent with a buffalo chest or a single chest cavity without anatomic separation of the two hemithoraces on computed tomography of the chest. Differential diagnoses for bilateral pneumothorax include pneumomediastinum and subcutaneous emphysema [11], neither of which was seen in the current patient.

## Conclusions

The present case highlights the importance of recognizing that not only ipsilateral but also contralateral pneumothorax may develop during pacemaker implantation by unilateral subclavian vein cannulation for various reasons, such as electrode perforation of the atrial wall, intrapleural communication (i.e., buffalo chest), and pneumomediastinum. Further clinical research is warranted to reduce the risk of this complication.
